# Clean Transformation of Ethanol to Useful Chemicals. The Behavior of a Gold-Modified Silicalite Catalyst

**DOI:** 10.3390/molecules21030379

**Published:** 2016-03-19

**Authors:** Ermelinda Falletta, Michele Rossi, Joaquim Henrique Teles, Cristina Della Pina

**Affiliations:** 1 Dipartimento di Chimica, Università degli Studi di Milano and CNR-ISTM, via Golgi 19, Milano 20133, Italy; ermelinda.falletta@unimi.it (E.F.); michele.rossi@unimi.it (M.R.); 2 Process Research and Chemical Engineering, BASF SE, Ludwigshafen 67056, Germany; henrique.teles@basf.de

**Keywords:** catalysis, gold, ethanol, acetaldehyde, acetic acid

## Abstract

Upon addition of gold to silicalite-1 pellets (a MFI-type zeolite), the vapor phase oxidation of ethanol could be addressed to acetaldehyde or acetic acid formation. By optimizing the catalyst composition and reaction conditions, the conversion of ethanol could be tuned to acetaldehyde with 97% selectivity at 71% conversion or to acetic acid with 78% selectivity at total conversion. Considering that unloaded silicalite-1 was found to catalyze the dehydration of ethanol to diethylether or ethene, a green approach for the integrated production of four important chemicals is herein presented. This is based on renewable ethanol as a reagent and a modular catalytic process.

## 1. Introduction

Over the last decades, ethanol (“bioethanol”) has emerged as a promising renewable feedstock available from carbohydrate biomass, thus providing alternative routes towards chemicals currently derived from fossil resources. The annual production of bioethanol, exceeding 50 million tons and steadily increasing, represents a sound raw material basis for various industrial applications. Henry Ford was one of the first to apply vegetable fuels (in particular, ethanol) for transport and the production reached 18 million tons/year in the 1930s at plants in Kansas. After the Second World War interest markedly decreased, owing to the huge availability of oil and gas. The first oil crisis of the 1970s aroused attention again on bioethanol as an alternative fuel source, as well as an ecological additive in gasoline. The past few years have seen a veritable boom in the advocacy and usage of bioethanol as a fuel, due to environmental concerns over global warming and promising oil-importing countries a relative independence from oil-exporting ones. As a consequence, bioethanol in the transportation sector has been subject to several studies and much discussion [1,2,3,4]. Its potential to mitigate greenhouse gases (*i.e.*, CO_2_ emissions) and replace fossil fuel-based oil represents the main reason why bioethanol is nowadays considered and implemented, but this is fully dependent on its production method. Bioethanol can be obtained by fermenting sugars contained in a wide range of biomass resources, each one differently effective at reducing carbon dioxide emissions and replacing fossil fuels. The main kinds of biomass are sugar-rich biomass (*i.e.*, sugar beet and sugarcane), starch-rich biomass (*i.e.*, grain, potatoes, sorghum, and cassava), and cellulose-rich biomass (*i.e.*, straw, wood, corncobs, stalks, grass, and paper). Presently, the most efficient way to achieve ethanol is via Brazilian sugar crops (namely, sugarcanes and beets). By the way, Brazil was the first and largest producer of bioethanol, but the USA, China, India, and Europe have recently increased their production as well. Starch crops represent the majority of the remaining feedstock, whereas cellulose-rich biomass is not yet commercially exploited [4]. The fermentation of sugars leads to watery ethanol, which requires distillation to concentrate the wet ethanol up to 95%, eventually followed by dehydration of the remaining 5% water to make fuel-grade ethanol. Despite some concerns related to biomass processing to bioethanol, *in primis* the food *vs.* fuel problem due to land use conflicts, bioethanol seems to be the best alternative to fossil fuel. Although the transportation sector represents the largest application area, a strategic usage of bioethanol is now emerging. Due to its increasing production and ensuing lower cost, bioethanol as a renewable feedstock could pave the green way to fundamental chemicals so far achieved via the petrochemical route.

**Figure 1:**
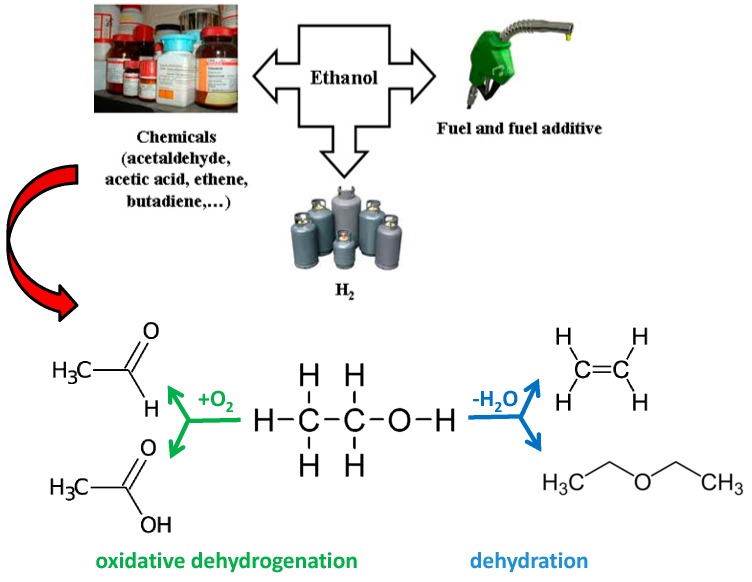
Possible uses of ethanol.

In addition to conventional dehydration reactions leading to diethyl ether or ethene, the catalytic oxidative dehydrogenation and oxidation of ethanol could serve as eco-friendly routes for the production of chemicals of interest (Figure 1).

**Figure 2:**
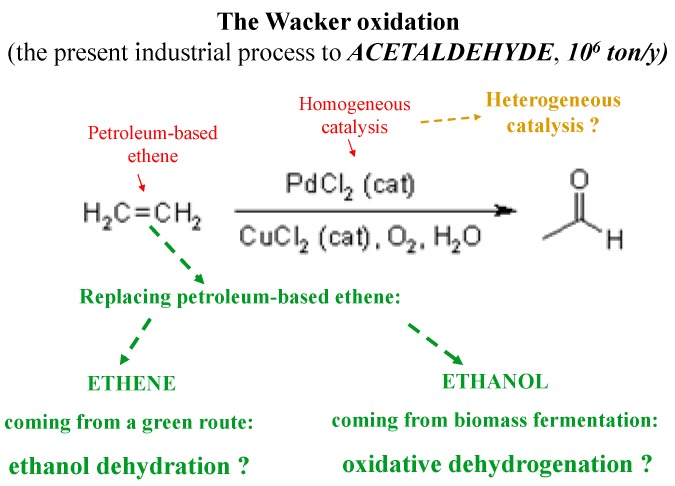
Potential green alternative to the Wacker oxidation.

In particular, these might provide sustainable alternatives to the Wacker oxidation of ethene in the production of acetaldehyde (Figure 2) or to methanol carbonylation to achieve acetic acid, but it can make sense where cheap ethanol is available.

The largest use for acetic acid is the manufacture of vinyl acetate monomer (VAM), which accounts for one-third of its consumption. Many processes have been used commercially for acetic acid production and such a technology is, perhaps, the most diverse among all the major industrially employed methods. No other large volume chemical can claim the varied feedstocks and production approaches as acetic acid can [5]. Even though the first production way was the aerobic fermentation of ethanol [6,7,8], methanol carbonylation has rapidly become the dominant technology accounting for about 80% global capacity and representing one of the most successful industrial scale applications of organometallic catalysis by transition metal complexes [9,10]. After the initial cobalt-based process commercialized by BASF, further advances in terms of activity and selectivity were reached by Monsanto with rhodium and iridium-based catalysts, a process operating for more than 40 years up to the introduction of Cativa™. In the 1990s, in fact, a promoted iridium/iodide-catalyzed methanol carbonylation process was introduced by BP Chemicals (the Cativa™ process) and is still active worldwide [11]. In spite of numerous advantages compared to the rhodium-based process, such a technology has not yet been able to depress the significant side reaction (the water-gas-shift reaction) which both of the processes suffer from. The principles of “green chemistry”, invoked to employ renewable materials and environmentally-benign solvents, have favored a renewed interest in bioethanol as a starting material for achieving acetaldehyde by catalytic dehydrogenation, eventually oxidized to acetic acid. Currently, ethanol is already the second largest feedstock for the production of acetic acid accounting for about 10% world capacity. While, as mentioned, the ethanol fermentation approach has been already explored during the last century, new biocatalytic routes to acetic acid are presently being experimented but the limits of fermentation process still remain [12]. The vapor phase oxidation or oxidative dehydrogenation of ethanol seems to offer a viable alternative due to a wide spectrum of effective catalysts (*i.e.*, ThMo_2_O_8_, Mo/meso TiO_2_, MgCrO, Zn-MCM-41, Cu-MCM-41, MgCrO, and CuCr_2_O_4_) [13,14,15,16,17,18]. The use of oxidants, such as dioxygen or air, reduces both cost and environmental impact [19], whereas the benefits provided by the gaseous phase compared to the liquid phase processes are well known in terms of efficiency improvement and no solid-liquid separation.

The first catalysts used for alcohol oxidation consisted of dispersed noble metal clusters and their oxides (e.g., Pt [20], Pd [21], and Ru [22]). Those based on palladium and platinum have recently drawn great attention for ethanol electro-oxidation in fuel cells [23]. Gold as a catalyst is a relatively novel discovery, which goes on delivering notable achievements in terms of performance and range of applications [24,25,26,27,28,29,30,31,32,33,34,35,36,37,38,39,40,41,42,43,44,45,46,47,48,49,50,51,52,53,54,55,56,57]. Considering further peculiarities, like biocompatibility, availability and ease of recovery, gold definitely appears to be a proper catalyst for sustainable processes based on the use of clean reagents under mild conditions, employing O_2_, air, or H_2_O_2_ as the oxidants, often in aqueous solution or in the absence of any solvent. Compared to other catalysts, mainly the platinum group metals, the most peculiar property of gold catalysis is the high selectivity which allows to discriminate between functional groups and geometrical positions, therefore leading to superior yields towards the desired products [34,37,47]. Hence, glycols could be oxidized to monocarboxylates [24,34,37,47] and unsaturated alcohols to unsaturated aldehydes [26,34,37,47]. Gold is active in catalyzing reduction reactions as well. Accordingly, unsaturated aldehydes and ketones could be hydrogenated to unsaturated alcohols with selectivity approaching 100% [32]. It has been recently observed that gold nanoparticles act as effective catalysts even in the oxidative polymerization of aniline and pyrrole to the corresponding conducting organic polymers (polyaniline and polypyrrole) [35,36,40]. A breakthrough in gold catalysis is the possibility of addressing selectivity to carboxylic acids or to aldehydes and ketones when aliphatic alcohol oxidation is performed in the liquid- or gas-phase, respectively [24,26,34,35,37]. Gold catalysis is dominated by heterogeneous catalysts, whereas homogeneous catalysts (the substrate and the catalyst are in the same state) still represent the smaller portion [42,43]. Actually, homogeneous gold catalysts are not capable of oxidizing alcohols with dioxygen; only the oxidation with hydroperoxides as oxidants to form esters was possible, as reported by Hashmi *et al.* [43].

In this kaleidoscopic context, ethanol conversion to valuable chemicals is rapidly gaining a prominent place. Some recent papers reported notable results in liquid-phase oxidation of ethanol towards acetic acid and acetyl acetate under air or dioxygen by heterogeneous gold catalysis (namely, Au/MgAl_2_O_4_, Au/TiO_2_, and Au/SiO_2_) [31,33,38]. In the vapor phase, as expected, the same reaction mainly yielded acetaldehyde with Au/CeO_2_, Au/SiO_2_, and Au/MgCuCr_2_O_4_ as the catalysts [28,41,49]. In particular, Hensen *et al.* recently found gold nanoparticles supported on MgCuCr_2_O_4_–spinel are highly active and selective for the aerobic oxidation of ethanol to acetaldehyde (conversion 100%; yield ∼95%) [49]. The flourishing of papers on gold catalysis, however, has not yet found a match in the patent literature [53,54,55,56,57]. This is a sign that further optimization both in catalyst design and process engineering is required.

In a former research, we have investigated the versatility of a robust catalyst derived from silicalite-1 (a MFI-type zeolite), which resulted in being effective in converting ethanol to diethyl ether or ethene with high selectivity by simply changing the catalyst pre-treatment and reaction conditions [44]. Following this latter investigation, herein we present novel advances in the selective conversion of ethanol by employing gold-modified silicalite-1 catalysts.

## 2. Results and Discussion

### 2.1. Catalytic Tests

#### 2.1.1. Ethanol Dehydration to Ethene and Diethyl Ether

In a previous work we have reported that silicalite-1 is activated towards dehydration reactions by acidic treatment (HCl) and inhibited by alkaline treatment (CH_3_COOK) [44]. The catalysts prepared as reported in Section 3.2 were tested in a continuous flow unit made up of a vertical glass reactor as detailed in Section 3.3. The results are summarized in Table 1.

The vapor phase conversion of ethanol over undoped and unloaded silicalite-1 (cat A, thermal treatment under air at T = 350 °C) started only at relatively high temperature (300 °C) addressing selectivity to diethyl ether Et_2_O (82%) at a modest conversion rate (7%). The higher temperature, 400 °C, allowed a 79% conversion while inverting selectivity to ethene C_2_H_4_ (98%). Doping silicalite-1 with diluted HCl (cat B) led to 98% selectivity towards Et_2_O at 39% conversion and at a low temperature of 240 °C. Increasing the temperature up to 300 °C shifted selectivity to 100% C_2_H_4_ at full conversion.

Moreover, cat B displayed steady stability for a long time, whereas XRPD (X-ray powder diffraction) and SEM (scanning electron microscopy) analyses confirmed morphology retention before and after use (T = 300 °C, 48 h). When silicalite-1 was doped with concentrated HCl (cat C), the catalytic performance remained unchanged at higher temperature (full conversion and 100% selectivity to the dehydration product C_2_H_4_ at 300 °C). Regarding the lower temperature, a benefit in terms of conversion was observed when compared to cat B, but at the expense of selectivity towards Et_2_O (82% selectivity, 58% conversion at 240 °C). In order to evaluate how alkali might affect the catalytic activity, silicalite-1 was impregnated with a base (CH_3_COOK) and the catalyst labeled as cat D. A marked depressing effect on the catalytic performance was registered since no ethanol conversion occurred at 300 °C. A slight improvement was detected by increasing temperature up to 400 °C. In this latter case, however, 42% selectivity to acetaldehyde CH_3_CHO (via oxidative dehydrogenation), besides 56% C_2_H_4_, is far from negligible. This might indicate a promoting effect of alkali over oxidative dehydrogenation to the detriment of dehydration. As further proof of alkali inhibition, cat B was recovered after 2 h on stream at 300 °C, doped with base (cat E) and then tested. Actually, the original activity of cat B was lost since cat E was unable to convert ethanol beyond 5% conversion at 300 °C. Interestingly, when cat E was re-doped with acid (cat F) the initial performance displayed by cat B was completely restored, thus underpinning its reversible acid activation-base deactivation. The concert of analytical techniques employed for finding a correlation between textural-morphological properties of the samples, and their catalytic activity indicated only weak and contrasting differences without a logical relationship (namely, TGA thermogravimetric analysis, N_2_ adsorption and desorption isotherms interpreted by BET and t-plot models, and EDS electron diffraction spectroscopy). Accordingly, hydrogen chloride might allow the formation of surface-protonated groups of Si-OH which, together with the neutral Si-O-Si bridges, enhance the final catalytic performance taking part in ethanol dissociative adsorption [44].

#### 2.1.2. Ethanol Oxidative Dehydrogenation to Acetaldehyde and Oxidation to Acetic Acid

**Figure 3:**
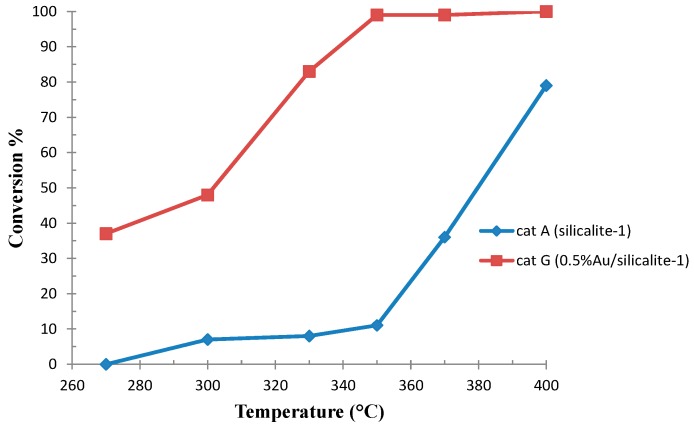
Comparison between unloaded silicalite-1 (cat A) and 0.5% Au/silicalite-1 (cat G) in ethanol conversion to Et_2_O and C_2_H_4_.

In order to address ethanol conversion towards acetaldehyde (CH_3_CHO) and acetic acid (CH_3_COOH) (via oxidative dehydrogenation and oxidation), we took inspiration from our experience in gold catalysis [24,26,27,34,36,40,44,47,52]. A series of Au/silicalite-1 catalysts were prepared, as reported in Section 3.2, and tested, as detailed in Section 3.3. By impregnating silicalite-1 (cat A) with a small amount of gold (0.5% Au/silicalite-1, cat G), the catalytic effect on ethanol conversion to Et_2_O resulted in being enhanced with respect to cat A. Accordingly, in the absence of oxygen, the reaction carried out with cat G started at a lower temperature than with unloaded silicalite-1, cat A (270 °C *vs.* 300 °C), reaching 90% selectivity to diethyl ether at 37% conversion (Table 2 exp 1 and Table 1, respectively). When the temperature was increased up to 400 °C, selectivity shifted to ethene with 96% selectivity (Table 2 exp 6). At low temperature (300 °C), and in the presence of a limited amount of oxygen in the stream (molar ratio O_2_/EtOH = 0.3), no effect on the product distribution was detected, whereas a strong thermal effect in the range 300 °C–330 °C was evident (Table 2 exp 2,3). The selectivity changed from 77% Et_2_O at 300 °C to 91% C_2_H_4_ at a temperature only 30 °C higher, along with a marked conversion increase (from 48% to 83%) (Figure 3).

Comparing unloaded silicalite-1 to 0.5% Au/silicalite-1 under the same experimental conditions, the beneficial effect of gold on conversion was not accompanied by an improved selectivity towards acetaldehyde or acetic acid, because the dehydration reaction dominates even with gold. As reported in [44] and mentioned in Section 2.1.1, silicalite-1 is activated towards dehydration reactions by acidic treatment (HCl) and inhibited by alkaline treatment (CH_3_COOK). This lets us surmise the acidic nature of silicalite-1, perhaps emphasized by the residual acidity of HAuCl_4_ (the gold source), which might be responsible for the unexpected acid-activated gold catalyst. Being a strong Brönsted acid, silicalite-1 by itself could act as a strong acid but, once treated with potassium acetate, could lose its activity for the formation of diethyl ether and ethene. In order to remove the masking effect of the acidic component, the residual acidity was buffered with various amounts of potassium acetate. The addition of CH_3_COOK/Au = 10, 20, 50 (molar ratio) gave rise, respectively, to cat H, I, and L.

Irrespective of the CH_3_COOK/Au ratio, and despite low ethanol conversion, total selectivity to acetaldehyde was maintained by operating in the absence of O_2_ (Table 3 exp 7, 9, 11, 13, and 15). Interestingly, at a temperature of 270 °C the catalytic activity progressively improved as the molar ratio CH_3_COOK/Au decreased, changing from the modest 4% conversion with cat L to 40% with cat H (Table 3 exp. 11, 9 and 7). The introduction of dioxygen promoted both ethanol conversion and acetic acid formation, making selectivity more sensitive to alkaline doping of the catalysts. Furthermore, a moderate amount of acetate able to buffer the catalytic surface acidity (cat I, CH_3_COOK/Au = 20) produced the best compromise between conversion and selectivity to acetaldehyde (97% at 71% conversion at 270 °C, Table 3 exp 10). Conversely, a large excess of acetate (Cat L, CH_3_COOK/Au = 50) worsened ethanol conversion and acetaldehyde production to the benefit of acetic acid formation (Table 3 exp 12).

Such data suggest a viable way to tune 0.5% Au/silicalite-1 performance by slightly neutralizing the acidic component of the catalyst and introducing a sub-stoichiometric amount of dioxygen. Accordingly, when operating without O_2_ in the temperature range 270 °C–350 °C, total selectivity to the dehydrogenation product CH_3_CHO was always obtained with all the catalysts, whereas cat G (no alkaline treatment) favored the dehydration products diethyl ether at the lowest temperature and ethene at the highest one. Most important, dioxygen acted both as a conversion enhancer and acetic acid promoter. Further experiments were carried out to evaluate how gold loading and dioxygen/ethanol ratio could influence the catalytic performance. The amount of gold was varied up to 16.3% wt. and O_2_/EtOH molar ratio from 0.5 to 1.5. The catalyst with the highest gold loading (16.3% wt.) and CH_3_COOK/Au = 20 (cat M) turned out to be the most promising (Table 4). Ethanol could be oxidized to the desired acetic acid with total conversion and high selectivity (79%), along with other commercially valuable products as ethyl acetate AcOEt (11%) and acetaldehyde (6%) at a relatively low temperature (250 °C) by using dioxygen in stoichiometric amounts.

**Figure 4:**
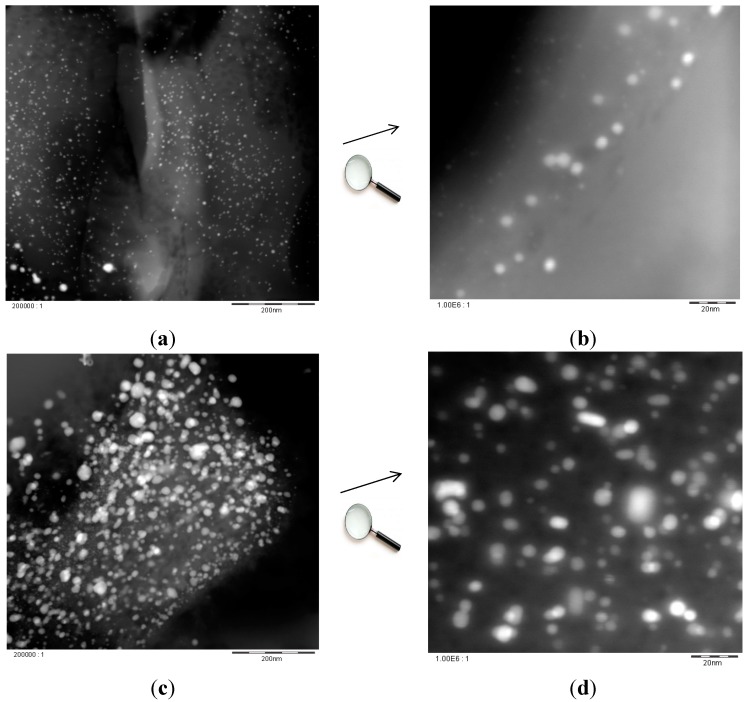
HAADF-STEM images of Au/silicalite-1 catalysts: (**a**) cat I (0.5%Au/silicalite-1 doped with CH_3_COOK/Au = 20), enlargement 200,000:1; (**b**) cat I, enlargement 1,000,000:1; (**c**) cat M (16.3% Au/silicalite-1 doped with CH_3_COOK/Au = 20), enlargement 200,000:1; and (**d**) cat M, enlargement 1,000,000:1.

As reported in Section 2.2 (Figure 4c,d), gold nanoparticles present in this high-loaded catalyst were observed both as single particles or clustered/agglomerated ones. The metal nanoparticles size is too polydispersed to conclude whether the dramatic increase in acetic acid formation and the gain in activity are due to the contribution of the large particles, not present in the low-loaded sample. However, some papers have reported that gold can be catalytically active even when displaying large size [26,39,45,46].

### 2.2. Catalyst Characterization

Silicalite-1 employed in this research has been extensively characterized in our previous paper [44]. Figure 4 visualizes HAADF-STEM (high angle annular dark field-scanning transmission electron microscopy) images for the most representative catalysts in ethanol conversion to acetaldehyde and acetic acid: cat I (0.5% Au/silicalite-1 doped with CH_3_COOK/Au = 20) and cat M (16.3% Au/silicalite-1 doped with CH_3_COOK/Au = 20).

The probes were first embedded in a polymer matrix for their fixation and then underwent “microtomy” by ultrathin slicing (separated by water flotation). In order to get a better contrast of the Au particles on the Si/O containing support material, the dark field method was used (HAADF-STEM). With this technique heavy elements and high-density materials are shown as bright spots, while the support appears to be in grey.

Regarding 0.5% Au/silicalite-1 (cat I), on the Si/O-particles, small, globular, crystalline Au particles with diameters between 1 and 15 nm could be observed. Sporadically, larger particles with up to 50 nm diameter could also be detected (Figure 4a,b). As to 16.3% Au/silicalite-1 (cat M), on the Si/O particles, more concentrated Au particles were obviously found when compared to cat I. The crystalline Au particles were observed both as single particles, but also in slightly clustered/agglomerated form. The particle size varied in a wide interval: whereas particles with *ca.* 1 nm were present; others with up to 1 µm were also visible (Figure 4c,d).

## 3. Materials and Methods

### 3.1. Reagents and Instruments

Commercial high-purity ethanol (95% *v/v*) and deionized milliQ water were used in the experiments. All of the gases (99.99%) were from SIAD, gold sponge (Fluka, 99.999%), silicalite-1 (Silikalite-1, 1 mm × 4 mm pellets, Si = 44%, Al < 0.01%, Fe < 0.005%, pore volume 0.33 mL g^−1^) was provided by BASF (Ludwigshafen, Germany). Gas-chromatographic analysis was carried out using online gas chromatography (HP5890 II) equipped with a HP-PLOT Q column (30 m × 0.53 mm × 40.0 µm film thickness) and a thermal conductivity detector (TCD). Helium was used as the carrier gas and nitrogen (4.76 mol %) as an internal standard. TEM images of the catalysts, 0.5% Au/silicalite and 16.3% Au/silicalite, were pictured by FEG-TEM (field emission gun-transmission electron microscopy), HAADF-STEM (high angle annular dark field-scanning transmission electron microscopy), EDXS (energy dispersive X-ray spectroscopy (Z > 8). TEM analyses were performed at BASF (Ludwigshafen, Germany), the rest of characterization analyses were carried out at Milan University, Italy.

### 3.2. Catalyst Preparation

Silicalite-based catalysts were prepared by impregnation method (“incipient wetness impregnation”) according to protocols similar to those reported in [44]:

- **cat A**: 3 g silicalite-1 was heated under air at 350 °C for 4 h;
- **cat B**: as cat A but impregnated with diluted HCl (0.03 mmol/g);
- **cat C**: as cat A but impregnated with concentrated HCl (3 mmol/g);
- **cat D**: as cat A but impregnated with CH_3_COOK·3H_2_O (0.3 mmol/g);
- **cat E**: cat B after 2 h on stream at 300 °C, thereafter impregnated with CH_3_COOK·3H_2_O (0.3 mol/g);
- **cat F**: cat E after 2 h on stream at 300 °C, thereafter impregnated with HCl (0.3 mmol/g);
- **cat G**: 3 g of cat A were impregnated with 1 mL HAuCl_4_ solution (Au = 15 mg/mL) thereafter heated under air at 350 °C for 2 h to produce 0.5% Au/silicalite-1;
- **cat H**: as cat G but with the addition of 228 mg CH_3_COOK·3H_2_O into HAuCl_4_ solution (CH_3_COOK)/Au = 10, molar ratio);
- **cat I**: as cat G but with the addition of 517 mg CH_3_COOK·3H_2_O into HAuCl_4_ solution (CH_3_COOK)/Au = 20, molar ratio);
- **cat L**: as cat G but with the addition of 1293 mg CH_3_COOK·3H_2_O into HAuCl_4_ solution;
- **cat M**: as cat I but impregnated with 1 mL HAuCl_4_ solution (Au = 490 mg/mL) to produce 16.3% Au/silicalite-1.

### 3.3. Catalytic Test Apparatus

**Figure 5:**
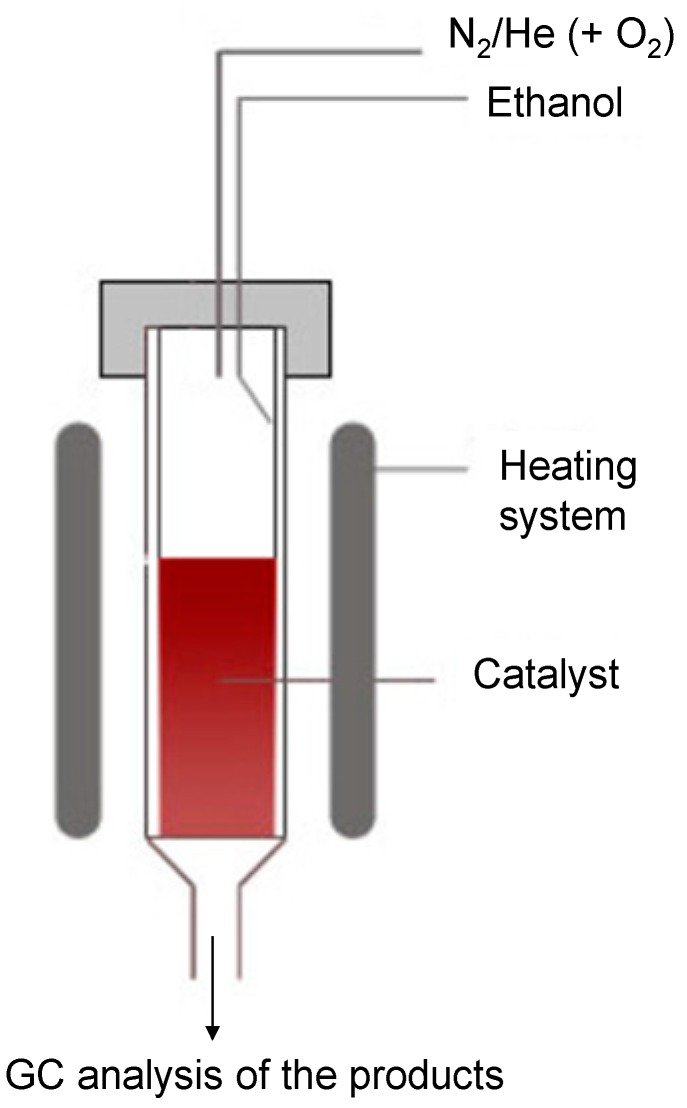
Vertical glass reactor.

All of the reactions were carried out in a continuous flow unit made up of a vertical glass reactor (*h* = 250 mm, *d* = 12 mm), fitted with a glass frit carrying the catalyst (3 g, *ca.* 4.2 mL) and provided with an electronically-controlled heating system. Air and nitrogen streams were controlled by mass flow instruments and the flow of the liquid reagent (95% ethanol) was supplied through an automatic syringe pump. Liquid vaporization occurred on the reactor wall prior to the catalytic bed. The tests were performed at different temperatures in the range of 250 °C–400 °C. The reactor exit was connected, by a thermostated line (180 °C), to the gas chromatograph injection port for the analysis of the products (Figure 5).

## 4. Conclusions

The experience matured during a former investigation on differently-doped silicalite-based catalysts was fundamental for dictating selectivity of ethanol conversion towards the oxygenated products, acetaldehyde, and acetic acid. By tuning the reaction conditions, adopting a specific surface treatment with potassium acetate and controlling gold loading, we were able to achieve 97% acetaldehyde selectivity at 71% conversion and 78% acetic acid at total conversion.

Amazingly, acetic acid was obtained over a catalyst having gold present as large particles with a wide size distribution. Although some researchers have found that even large gold particles can be catalytically active, in our case the metal size is too polydispersed to conclude whether the marked increase in acetic acid formation and the gain in activity are due to the contribution of these large particles. Gold supported on silicalite-1 catalysts may offer a promising eco-friendly way to acetic acid and/or acetaldehyde production that, once optimized, could turn out to be a sustainable alternative to the presently applied methods. Furthermore, having previously found silicalite-derived catalysts able to transform ethanol into diethyl ether or ethene with high selectivity by simply changing the reaction conditions and acidic doping, this modular catalytic process could pave the green way to fundamental chemicals so far achieved via the petrochemical route.

## Figures and Tables

**Table 1:** Ethanol dehydration on silicalite-1-based catalysts.

Catalysts	Cat A		Cat B		Cat C		Cat D		Cat E		Cat F	
**T** (°C)	300	400	240	300	240	300	300	400	300	400	240	400
**Conv.** %	7	79	39	100	58	100	0	10	5	42	36	100
**Sel Et_2_O** %	82	0	98	0	82	0	0	0	0	0	96	0
**Sel C_2_H_4_** %	18	98	2	100	18	100	0	56	100	68	4	100
**Sel CH_3_CHO** %	0	2	0	0	0	0	0	42	0	32	0	0

Cat A (thermal treatment under air at T = 350 °C), cat B (acidic doping with diluted HCl and thermal treatment under air at T = 350 °C), cat C (acidic doping with concentrated HCl and thermal treatment under air at T = 350 °C), cat D (alkaline doping with CH_3_COOK and thermal treatment under air at T = 350 °C), cat E (cat B after 2 h on stream at T = 300 °C, thereafter alkaline doping with CH_3_COOK and thermal treatment under air at T = 350 °C), and cat F (cat E after 2 h on stream at T = 300 °C; thereafter acidic doping with HCl and thermal treatment under air at T = 350 °C). EtOH = 27.5 mmol/h, He + N_2_ = 294.6 mmol/h, P = 1 atm, T = 240 °C–400 °C.

**Table 2:** Performance of cat G (0.5% Au/silicalite) in ethanol conversion.

Exp	O_2_/EtOH (mol ratio)	T (°C)	Conv. (%)	Selectivity (%)				
				CH_3_CHO	CH_3_COOH	CO_2_	Et_2_O	C_2_H_4_
1	0	270	37	6	0	0	90	2
2	0.3	300	48	2	0	0	77	8
3		330	83	2	0	0	7	91
4		350	99	2	0	0	0	98
5		370	99	3	0	0	0	97
6	0	400	100	4	0	0	0	96

EtOH = 27.5 mmol/h, He + N_2_ = 294.6 mmol/h, O_2_/EtOH (molar ratio) = 0–0.3, T = 270 °C–400 °C.

**Table 3:** Performance of cat H, I, and L (0.5% Au/silicalite-1 doped with CH_3_COOK. cat H = CH_3_COOK/Au = 10; cat I = CH_3_COOK/Au = 20, cat L = CH_3_COOK/Au = 50) in ethanol conversion.

Exp	Catalysts	O_2_/EtOH (Mol Ratio)	T (°C)	Conv. (%)	Selectivity (%)		
					CH_3_CHO	CH_3_COOH	CO_2_
7	H	0	270	40	100	0	0
8		0.3		68	86	13	1
9	I	0		19	100	0	0
10		0.3		71	97	2	1
11	L	0		4	100	0	0
12		0.3		46	72	21	7
13	I	0	300	41	100	0	0
14		0.3		75	86	12	2
15		0	350	54	100	0	0

EtOH = 27.5 mmol/h, He + N_2_ = 294.6 mmol/h, O_2_/EtOH (molar ratio) = 0–0.3, T = 270 °C–350 °C.

**Table 4:** Performance of cat M (16.3% Au/silicalite-1 doped with alkali, CH_3_COOK/Au = 20) in ethanol conversion.

O_2_/EtOH	T (°C)	Conversion %	Selectivity %			
			CH_3_CHO	CH_3_COOH	AcOEt	Others
0.7	250	100	24	61	13	2
1.0		100	6	79	11	4
1.5		100	4	78	9	9
0.5		95	53	28	18	1

EtOH = 27.5 mmol/h, He + N_2_ = 294.6 mmol/h, O_2_/EtOH (molar ratio) = 0.5–1.5, T = 250 °C.
